# A theory-informed, rapid cycle approach to identifying and adapting strategies to promote sustainability: optimizing depression treatment in primary care clinics seeking to sustain collaborative care (The Transform DepCare Study)

**DOI:** 10.1186/s43058-022-00383-2

**Published:** 2023-01-25

**Authors:** Nathalie Moise, Alejandra Paniagua-Avila, Jennifer Mizhquiri Barbecho, Luis Blanco, Katherine Dauber-Decker, Samantha Simantiris, Martin McElhiney, Maria Serafini, Darlene Straussman, Sapana R. Patel, Siqin Ye, Andrea T. Duran

**Affiliations:** 1 Department of Medicine, Center for Behavioral Cardiovascular Health, Columbia University Irving Medical Center, New York, NY USA; 2 Mailman School of Public Health, Columbia University, New York, NY USA; 3 Northwell Health, New York, NY USA; 4 The New York State Psychiatric Institute, New York, NY USA; 5 Vagelos College of Physicians and Surgeons, Columbia University, New York, NY USA

## Abstract

**Background:**

Few real-world examples exist of how best to select and adapt implementation strategies that promote sustainability. We used a collaborative care (CC) use case to describe a novel, theory-informed, stakeholder engaged process for operationalizing strategies for sustainability using a behavioral lens.

**Methods:**

Informed by the Dynamic Sustainability Framework, we applied the Behaviour Change Wheel to our prior mixed methods to identify key sustainability behaviors and determinants of sustainability before specifying corresponding intervention functions, behavior change techniques, and implementation strategies that would be acceptable, equitable and promote key tenets of sustainability (i.e., continued improvement, education). Drawing on user-centered design principles, we enlisted 22 national and local stakeholders to operationalize and adapt (e.g., content, functionality, workflow) a multi-level, multi-component implementation strategy to maximally target behavioral and contextual determinants of sustainability.

**Results:**

After reviewing the long-term impact of early implementation strategies (i.e., external technical support, quality monitoring, and reimbursement), we identified ongoing care manager CC delivery, provider treatment optimization, and patient enrollment as key sustainability behaviors. The most acceptable, equitable, and feasible intervention functions that would facilitate ongoing improvement included environmental restructuring, education, training, modeling, persuasion, and enablement. We determined that a waiting room delivered shared decision-making and psychoeducation patient tool (DepCare), the results of which are delivered to providers, as well as ongoing problem-solving meetings/local technical assistance with care managers would be the most acceptable and equitable multi-level strategy in diverse settings seeking to sustain CC programs. Key adaptations in response to dynamic contextual factors included expanding the DepCare tool to incorporate anxiety/suicide screening, triage support, multi-modal delivery, and patient activation (vs. shared decision making) (*patient*); pairing summary reports with decisional support and yearly onboarding/motivational educational videos (*provider*); incorporating behavioral health providers into problem-solving meetings and shifting from billing support to quality improvement and triage (*system*).

**Conclusion:**

We provide a roadmap for designing behavioral theory-informed, implementation strategies that promote sustainability and employing user-centered design principles to adapt strategies to changing mental health landscapes.

**Supplementary Information:**

The online version contains supplementary material available at 10.1186/s43058-022-00383-2.

Contributions to the literature

- This study provides a novel roadmap for leveraging behavior and implementation science as well as user-centered design principles to design and rapidly adapt theory-informed strategies for sustainability.
- In addition to external support, sustaining collaborative care may require ongoing patient activation, provider decisional support/motivation, and local technical assistance/problem-solving.
- User-centered design principles may help operationalize the need for continuous improvement/adaptation in the sustainability phase.

## Background

More than 100 randomized trials demonstrate that collaborative care (CC), a team-based approach to managing depression in primary care, improves access to mental healthcare; improves depression, quality of life, and productivity; and is cost-effective compared to usual care by primary care providers (PCPs) alone [1–3]. CC is also one of the few evidence-based interventions shown to reduce health disparities [4], with minorities experiencing better improvements in depressive symptoms, physical and/or mental functioning, and unmet treatment needs [5] as well as receipt of preferred depression treatment [6]; and reduced perceived racial discrimination [7].

CC expanded exponentially across the USA, facilitated by Patient Centered Medical Home initiatives, Accountable Care Organizations, and new reimbursement approaches as part of primary care redesign [8] and through the Centers for Medicare and Medicaid Services [9]. Several recent initiatives suggest that implementation strategies such as providing flexible funding for staff, training, monitoring, quality improvement, and external facilitation improve CC implementation [10–13]. Unutzer et al. (2020) recently found that external support is essential to CC implementation and real-world effectiveness [14]. However, few studies focus on how best to select, refine and adapt strategies for CC sustainability, which has been defined as “the continued use of program components and activities for the continued achievement of desirable program and population outcomes” [15]. Here, we defined CC sustainability as *continued* provider CC referral/treatment optimization, patient CC enrollment/attendance, and care manager delivery of registry-based, treat to target depression treatment with population-based psychiatry consultation.

In 2015, building upon years of experience implementing CC for depression, the New York State (NYS) Department of Health (DOH) and NYS Office of Mental Health (OMH) successfully implemented CC in diverse, low socioeconomic status settings using a strategy consisting of technical assistance, fee-for-quality reimbursement, and ongoing training/quality monitoring; the program has reached more than 300 primary care clinics across the state [16]. In a mixed-methods study of 32 clinics about 4 or more years post-implementation, we demonstrated that having a full-time depression care manager dedicated to CC and early success (i.e., average improvements in depressive symptoms of 50% in the initial 2 years of implementation) determined whether a clinic would sustain CC or opt-out [17]. We found that external technical assistance and financial resources resulted in long-term fidelity and clinical improvement, but that clinics seeking to sustain CC often encountered low patient, staff (e.g., depression care managers), and PCP engagement as well as limited resources (e.g., number of depression care managers), perhaps due to implementation drift (e.g., decay in fidelity to depression screening) and turnover of program champions over time [17].

The NYS findings suggest that strategies designed for early implementation may not be sufficient for sustainability, particularly in diverse settings with the most to gain from the model. There is a recent focus on sustainability as a dynamic concept, recognizing that the implementation of evidence-based interventions will likely evolve over time, due to complex and changing real-world healthcare settings and systems [18, 19]. The Dynamic Sustainability Framework (DSF) argues for the continuous refinement and improvement of interventions during the sustainability phase, through learning and evaluation, problem solving and ongoing adaptations to interventions to enhance fit between interventions, practice settings/contexts and ecological systems over time [19]. Few guides exist for operationalizing this dynamic process, particularly the additions/revisions to initial *implementation strategies* that are essential to this process. In addition, the DSF does not explicitly acknowledge the ways in which sustainability often requires sustained *behavior change* at multiple levels (e.g., continued CC enrollment/attendance by patients). We posit that behavior change theory and user-centered design principles may be helpful tools for operationalizing the dynamic sustainability process.

The NYS DOH/OMH initiative for implementing CC for depression provides a unique, rare opportunity to identify how best to develop, adapt and test strategies for sustainability in real world settings. This paper provides a roadmap for designing and adapting behavioral theory-informed strategies for sustainability, drawing from the DSF, Behaviour Change Wheel (BCW) [20], Expert Recommendations for Implementing Change (ERIC) [21], and user-centered design principles [22–24]. To our knowledge, this is one of the first studies to describe the design of a multi-level, theory-informed, adaptable implementation strategy for settings seeking to sustain CC programs.

## Methods

### Synopsis of our prior work on implementing CC

Based on our prior mixed methods analyses [17], we determined that NYS OMH’s ongoing implementation and scale-up strategies (i.e., external technical assistance, quality monitoring, reimbursement) optimized CC uptake, quality/fidelity, clinical improvement/psychiatry consultation rates, acceptability, and perceived costs but that referral/enrollment rates (which are directly tied to billing and thus care manager hiring/retention) diminished significantly over time despite stable depression screen positive rates. We surmised that care manager, patient, and provider engagement were key to sustainability, which would require strategies that not only addressed changing local contextual factors (like problem-solving/adaptation) but also promoted ongoing multi-level behavior change.

### Overview

**Fig. 1:**
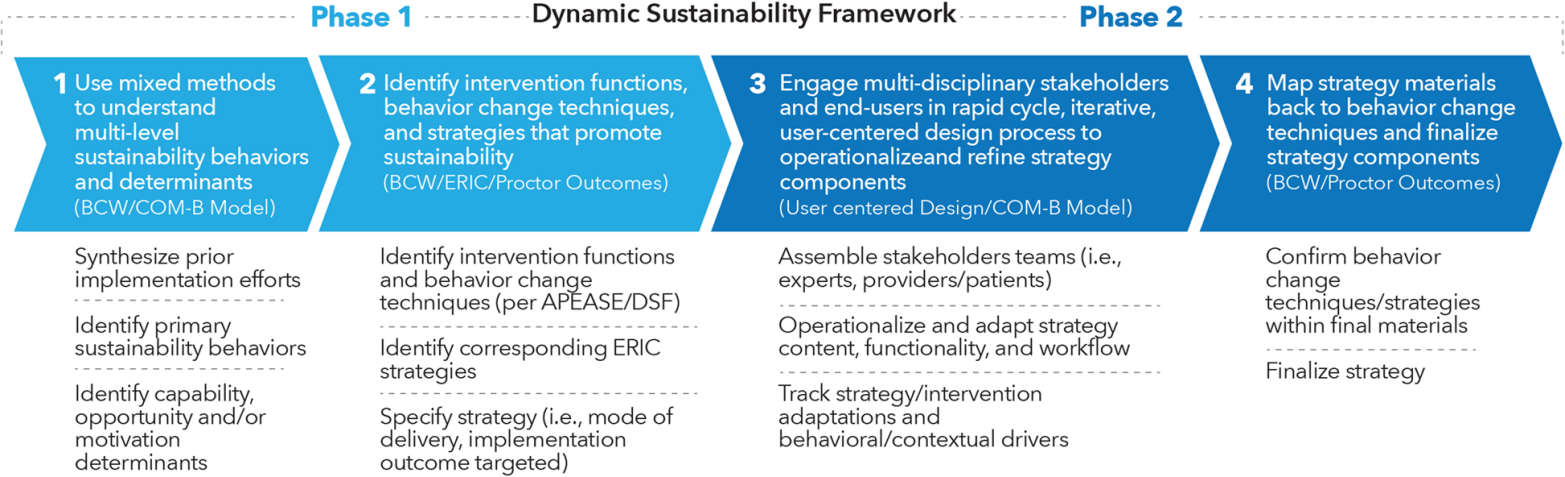
Roadmap for theory-informed approach to identifying and adapting strategies for sustainability

Informed by the DSF, our development of implementation strategies to enhance CC sustainability consisted of the following phases: (1) a mixed methods approach to specify determinants of and strategies for sustainability based on the multi-step BCW [20] and ERIC [21]; and (2) a rapid cycle adaption phase, drawing from user-centered design principles [22–24], that involved multidisciplinary stakeholders in operationalizing and refining a multi-level implementation strategy for sustainability to maximize fit between the intervention/CC, strategy and context over time (Fig. 1).

### Theoretical frameworks used to develop implementation strategies that promote CC sustainability

Theoretical underpinnings of our approach centers around the DSF (*process framework*), particularly the need for ongoing optimization, improvement, evaluation/feedback, stakeholder involvement, and organizational learning as well as a strong fit between the program and implementation setting, all of which we argue require sustained behavior change on the parts of multiple stakeholders.

For *Phase 1*, we used the BCW (Additional File 1), which can be applied to individual behavior (e.g., patient-level smoking cessation) and multi-level (system, provider, patient) behavior change in clinical settings [20], as well as implementation strategy development [25, 26]. The multi-step BCW first requires identifying and specifying a primary behavior and posits that changing behavior requires increasing capability, opportunity, and/or motivation for a behavior (COM-B Model) (*determinant framework*) by removing barriers and/or augmenting facilitators to that target behavior. The BCW involves (1) understanding the behavior, (2) identifying intervention options, and (3) identifying related content and implementation options [20]. Informed by the DSF, we sought to apply the BCW to understand behaviors integral to sustainability while identifying implementation strategies informed by both behavioral theory and dynamic sustainability tenets. We further characterized strategies according to ERIC [21] and targeted Proctor’s implementation outcome [27] (*outcome framework*) in order to facilitate standardized reporting across implementation science studies [21, 28].

**Fig. 2:**
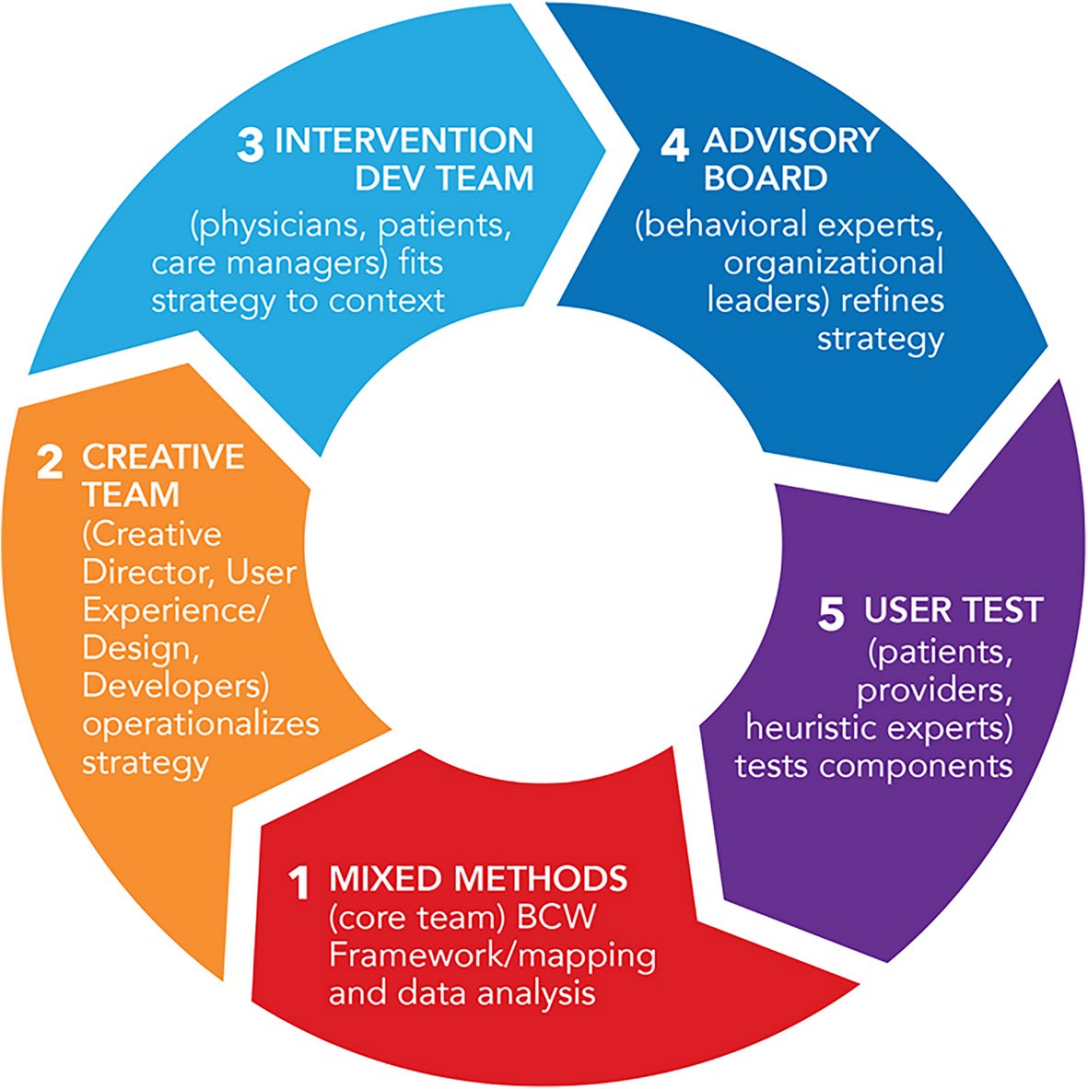
Rapid cycle, stakeholder engaged adaption of strategies for sustainability

For *Phase 2*, we aimed to operationalize a key tenet of DSF (i.e., adaptation to maximize the fit between CC, implementation strategies, and context over time) by using stakeholder engaged, rapid cycle process*.* We leveraged user-centered design principles, an approach to product development that grounds the process in data collected from end users at the individual and settings level; this process draws from a clear identification of the end users and their needs, prototyping/rapid iteration, simplification of existing procedures and exploiting natural constraints [22, 24, 29]. We specified behavioral (COM-B model) and contextual (DSF) factors driving adaptations. Here, we describe iterations made by the advisory board, intervention development team, and creative team and will describe patient-level usability testing, cognitive interviews, and heuristics evaluation separately (Fig. 2) [30].

### Setting

We developed the multi-level implementation strategy for sustaining CC in a large academic primary care clinic in the Ambulatory Care Network (ACN) at New York Presbyterian Hospital in upper Manhattan serving low socioeconomic and diverse communities of Washington Heights, Inwood, Harlem, and the Southwest Bronx. Implementation is currently occurring in separate demographically similar ACN clinics in the sustainability phase of the NYS OMH CC Initiative (i.e., receiving external technical assistance/quality monitoring and fee-for-quality reimbursement for Medicaid patients) as well as integrated care settings not receiving external implementation strategies.

### Phase 1: specify multi-level determinants of and strategies for CC sustainability

#### Step 1 methods: use mixed methods to understand multi-level sustainability behaviors and determinants

Overall, our work was informed by three previously published mixed-methods studies on sustainability of CC, including (1) descriptive analyses of CC sustainability including long-term fidelity, improvement, enrollment, caseloads, and psychiatric consultation rates as well as 30 semi-structured interviews with PCPs, care managers, psychiatrists and administrators at clinics that sustained or opted out of CC across in NYS [17]; (2) three focus groups and four one-on-one interviews with historically marginalized patients (*n*= 12) referred to CC programs in the sustainability phase (both enrollees and no-shows) [31]; and (3) 10 interviews with CC experts from diverse settings across the USA focused on determinants of patient engagement in mature (i.e., at least 1 year after implementation initiation) CC programs [32].

First, we defined the problem in behavioral terms and selected and specified target behaviors at every level (patient, provider, and system). Drawing from our previously published qualitative interviews with key stakeholders on barriers to CC sustainability (above) [17, 31, 32], two authors (NM, MAPA) classified themes highlighted in the publications by level (patient-, provider- or system-level) and COM-B construct. For example, we deemed the ‘lack of PCP time and competing demands’ theme to be a provider-level “opportunity” barrier. There were no major disagreements. We resolved any differences by consensus discussion, with researchers referring to original manuscripts to reassess context of the codes when categorizations were unclear.

#### Step 2 methods: identify intervention functions, behavior change techniques and strategies that promote sustainability

After coding the COM-B categories, our clinician implementation scientists on our research team (NM, MAPA) followed the BCW to map the above COM-B barriers to corresponding intervention functions (i.e., nine broad categories by which an intervention can change behavior, e.g., education, training, persuasion), policy categories (i.e., seven policies representing types of decisions made by authorities that help to support and enact interventions), and behavior change techniques (i.e., a standardized language for describing the active ingredients in behavior change interventions via which intervention functions and policy categories are delivered (Additional File 2). As several interventions, policies, and behavior change techniques may map to each COM-B construct, the BCW recommends applying the affordability, practicality, effectiveness, acceptability, side effects, and equity (APEASE) criteria to narrow-down intervention components to those that are affordable (within an acceptable budget for patients, mental health and primary care providers, and administrators after/not withstanding development costs), practical (can be delivered as designed), efficacious (effectiveness and cost-effective related to designed objectives in real world context), acceptable (judged appropriate by relevant stakeholders), safe (no unwanted side effects), and equitable (does not increase disparities) [20].

Informed by DSF, we selected intervention functions, policy categories, and behavior change techniques that met all APEASE criteria (agreed upon by both coders) in the *sustainability phase*. We then reviewed and prioritized implementation components that aligned with core DSF tenets, particularly those not otherwise delivered within ongoing external implementation strategies. For example, incentivization (or the expectation of reward for patients enrolling in CC) was an intervention function that would be acceptable, effective, and equitable but did not align with continuous improvement/problem-solving DSF tenets nor was it affordable in the sustainability phase (i.e., OMH already provided fiscal reimbursement to care managers in sustainability phase and OMH/local settings would be unable to also incentivize patients). As another example, several enablement-related behavior change techniques met all APEASE criteria, but *problem-solving* particularly adhered to DSF principles. Differences were resolved through consensus discussion.

Given calls for shared language and conceptual clarity around implementation strategies, we also mapped behavior change techniques to implementation strategies from the ERIC project [21, 33], again prioritizing those strategies that met key DSF tenets. While all strategies met all APEASE criteria (i.e., would ongoing provider education be acceptable to stakeholders?), we also specified the dose, mode of delivery and predominant Proctor implementation outcome targeted (would this educational strategy improve CC acceptability?) for each strategy component [28].

### Phase 2: rapid cycle adaptation of an implementation strategy to promote sustainability

#### Step 3 methods: Engage multi-disciplinary stakeholders and end-users in rapid cycle, iterative, user-centered design process to operationalize and refine strategy components

After initial implementation strategy selection described above, we conducted sequential group and one-on-one meetings with multidisciplinary teams of stakeholders (Fig. 2) from January 2018 to May 2021 (~ 30 months to account for COVID-19 disruptions): (1) a creative team led by our creative director of developers and experts in user experience who create journey maps of patient and system/provider experiences with CC/mental health optimization and operationalize/develop patient and PCP facing materials; (2) an intervention development team of historically marginalized patient stakeholders, care managers, and PCP stakeholders who ensured strategies fit to rapidly shifting contextual factors in diverse, low socioeconomic settings; (3) an advisory board of experts in behavior change and patient activation/experience to refine the strategy; and finally, (4) which will be described separately, depression care managers, PCPs and historically marginalized patients for user testing patient-facing interventions to maximize the usability, safety, feasibility, and sustainability of our multi-level strategy.

For intervention team stakeholders, we used snowball sampling to identify CC champions in the ACN clinics. We identified advisory board members from literature reviews on behavioral, user experience and shared decision-making experts. Stakeholders were invited by email to serve in an advisory role for our study. While the cycle initiated with creative team members and ended with patient/provider user testing, intervention development, and advisory board meetings were in no apparent order; team members could meet multiple times in a row (as a group or one-on-one) based on need/mandate/availability. Meetings were held in-person with remote stakeholders calling in via teleconference prior to COVID-19 and via zoom/video calls post-COVID-19. Patients and providers who underwent in-person user-testing (described separately) were consented and the protocol was approved by the Columbia University Irving Medical Center Institutional Review Board.

The core team of staff and implementation science researchers recorded and transcribed meeting notes, identified themes, and arrived at consensus to guide strategy prototype refinements when technically and logistically feasible. We rapidly reviewed meeting notes for usability themes (i.e., content, usability, usefulness, understandability/functionality, visibility, workflow, navigation, content) based on key user-centered design principles [34–36]. We specified whether strategy adaptations were done to maximally target behavioral determinants of sustainability (informed by the BCW/COM-B Model [i.e., *motivation*, *opportunity, and/or capability*]) and/or contextual factors (informed by DSF [i.e., *ecologic system*: other practice settings, policy, regulation, population characteristics; *practice setting*: staffing, info systems, organizational culture, training, supervision]). Because *strategy adaptations* are often in response to *intervention* adaptations, we also tracked adaptations to the CC program itself overtime based on input from key stakeholders during meetings (again specifying driving behavioral and contextual factors driving CC adaptations).

#### Step 4 methods: map strategy materials back to behavior change techniques and finalize strategy components

The creative team met after each cycle to refine educational materials before presenting prototypes to the advisory board and intervention team members for feedback, consensus, and adaptation. We conducted three iterative adaptation cycles. At the end of the last cycle, we ensured the mode of delivery/behavior change techniques fit the contextual considerations of the telemedicine/post-COVID-19 era (e.g., we expanded the mode of delivery from iPads in waiting rooms to include home delivery via personal phone, computer, or tablet). An external expert trained in the BCW with no prior knowledge of our intervention then coded our final multi-level implementation strategy components (e.g., shared decision-making tool, PCP marketing videos) for behavior change techniques to ensure that behavioral components determined in Phase 1 were well represented in our final materials.

In our final advisory board meeting, we administered the brief feasibility and appropriateness of intervention measures [37] informed by Proctor’s implementation outcomes to ensure the feasibility and appropriateness/fit of our multi-level implementation strategy. We used descriptive analyses of our quantitative implementation outcomes to report percentages for categorical variables and means for continuous variables.

## Results

### Step 1 results: understand multi-level sustainability behaviors and determinants

#### Sustainability behaviors

We identified sustained engagement in CC by care managers/psychiatrists (system-level), depression treatment optimization/referrals (provider-level), and treatment initiation/persistence (patient-level/primary sustainability behavior given reimbursement link) as essential behaviors for collaborative/integrated care sustainability in diverse settings (Additional File 3). We combined system and provider-level constructs given marked overlap in interviews and themes.

#### Determinants of sustainability behaviors

Determinants of sustainability behaviors are presented in Table 1. Key *capability* constructs included lack of patient awareness of depression treatment options and provider CC knowledge/training (i.e., indications for referral) and error-prone referral processes (e.g., inappropriate referral of patients with serious mental illness). From an *opportunity* perspective, competing initiatives, limited resources/complex psychosocial needs, complex workflows as well as PCP and care manager time/schedules/workloads were key provider/system level barriers, while stigma, accessibility, convenience, and quality of mental care were key patient-level barriers. Key provider/system level *motivation* barriers related to ongoing PCP engagement, lack of PCP-care manager teamwork/communication, and infeasible warm handoffs while patients faced fear of treatment side effects and concerns around treatment efficacy (e.g., due to prior treatment failure). Experts also noted unaddressed patient-level concerns and the need for tailoring (e.g., for Spanish speaking participants) in the sustainability phase.

**Table 1:** Capability, Opportunity, and Motivation Constructs of Behavior (COM-B) at the provider/system and patient levels

**COM-B construct**	**Provider/system level themes**	**Patient level themes**
Psychological capability	*PCP training/knowledge* -Lack of knowledge about CC *Error-prone referral processes*	*Patient education about treatment options* *Patient’s lack of awareness of their own mental health*
Social opportunity	*External environment* -Competing primary care initiatives -Restrictive enrollment requirements -Inadequate resources and complex psychosocial needs	*Stigma about mental health*
Physical opportunity	*Funding* -Complex funding streams -Insufficient funding *Information technology/infrastructure* -Antiquated data management/information technology infrastructure *PCP time/resources/personnel* -Time constraints on PCPs -Competing PCP demands -Inflexible PCP schedule -High PCP workload limits depression diagnosis and treatment *Complex workflows* -Infeasible warm handoffs in sustainability phase (also motivation barrier) -Complicated screening, referral, and triaging (also capability, motivation barrier) *DCM time/resources/personnel* -Competing DCM roles, insufficient DCMs/personnel -Inadequate space -Inflexible mental health workers’ schedule	*Treatment accessibility and convenience* *Providers’ expertise and quality of mental care*
Reflective motivation	*Provider engagement* -Lack of primary care physician pro-activeness -Poor continuity of care -Poor psychiatrist engagement -Lack of DCM engagement -Inadequate teamwork/communication *Workflow logistics* -Complicated screening, referral, and triaging (also opportunity, capability barriers) *Beliefs about consequences* -Inadequate PCP buy-in -PCP concern for the validity of measures	*Patients’ beliefs about treatment being ineffective* *Unaddressed patient needs and preferences* *Patient engagement/self-efficacy* -Depression treatment stigma -Patient non-adherence -Limited language/literacy/cultural beliefs of patients -Infeasible warm handoffs (also opportunity barrier)
Automatic motivation		*Patients’ fear of treatment*

*Abbreviations*: *CC* Collaborative care, *DCM* Depression care manager, *PCP* Primary care providers

### Step 2 results: intervention functions, behavior change techniques and strategies that promote sustainability

#### Intervention function behavior change techniques and ERIC strategies for sustainability

Tables 2 and 3 describe the operationalized patient and provider/system-targeted multi-component implementation strategy with the *final list* (see below for adaptations) of corresponding behavior change techniques, targeted COM-B constructs, intervention functions (corresponding DSF tenet), mode of delivery/actors and implementation outcomes targeted [27] (e.g., CC fidelity, acceptability, sustainability).

**Table 2:** Patient-targeted multi-component implementation strategy for promoting collaborative care enrollment: behavior change techniques, intervention functions, mode of delivery as well as targeted behavioral constructs and implementation outcomes

**Mode of delivery/actor** **Dose** **Implementation outcome targeted**	**Strategy**	**Behavior change techniques**	**Intervention function (aligned DSF tenet)**	**COMB** **construct**				
				Psych capability	Social opportunity	Physical opportunity	Reflective motivation	Automatic motivation
eSDM tool/brochure via iPad/phone/computer/mailer in waiting room/home delivered by text/email/in-person by implementation staff/navigator Once/patient visit Acceptability, usability, fidelity	Patient activation, psychoeducation, and referral with flexible modality (takes place of warm handoff) Connect to alternative treatment options/modalities (Provide therapy options by phone/online/video/self-help); Tool with Spanish language option, low literacy considerations	Adding objects to environment	Environmental Restructuring (*continued adaptation/improvement*)	X	X	X		X
eSDM Tool/brochure delivered by text/email/in-person by implementation staff/navigator Once/patient visit Fidelity, sustainability	eSDM tool prior to PCP visit to facilitate conversation, reminder of need for treatment	Prompts/cues	Environmental restructuring/education (*continued adaptation,* *improvement, learning)*	X	X	X	X	
eSDM Tool (*patient video/content hub*)/brochure delivered by text/email/in-person by implementation staff/navigator Once/patient visit Acceptability	English/Spanish minority patient modeling engagement, going to treatment, and impact on relationships/life; Information on how to engage in treatment, talk to provider/therapist	Demonstration of behavior, information about social and environmental consequences	Persuasion, modeling, education (*continued learning, improvement*)	X	X	X	X	X
eSDM Tool (*care manager video*)/brochure delivered by text/email/in-person by implementation staff/navigator Once/patient visit Acceptability	Care manager describes CC, provides psychoeducation and treatment options	Credible source	Persuasion *(continued learning, improvement)*		X	X	X	X
eSDM Tool (*patient/care manager video/content hub*)/brochure delivered by text/email/in-person by implementation staff/navigator Once/patient visit Acceptability	Care manager/patient/content hub discuss ability to do treatment/improve, motivational messaging, health consequences (e.g., pain)	Verbal persuasion about capability, Information about health consequences	Education, persuasion, enablement (*Continued learning)*	X	X		X	X

*Abbreviations CC* Collaborative care, *DSF* Dynamic Sustainability Framework, *eSDM* Electronic shared decision making, *PCP* Primary care providers

**Table 3:** PCP/system-targeted multi-component implementation strategy for promoting collaborative care enrollment: behavior change techniques, intervention functions, mode of delivery as well as targeted behavioral constructs and implementation outcomes

**Final mode of delivery/actor** **Dose** **Implementation outcome targeted**	**Strategy**	**Behavior change techniques**	**Intervention function (aligned DSF tenet)**	**COMB** **construct**				
Onboarding PCP video delivered via email by clinic administrator Once Acceptability, fidelity	Education about how to order/refer to CC appropriately (e.g., demonstrates ideal note), social/environmental effects (e.g., impact of inappropriate referrals on resources, wait times, impact on patient outcomes)	Instruction on how to perform behavior, Demonstration of behavior, information about social, environmental, and health consequences	Education, persuasion, training, modeling *(continued learning/improvement)*	X	X	X	X	X
EPIC Message by implementation staff and automated email from eSDM tool to PCP Once/patient visit Acceptability, fidelity, feasibility, sustainability	Summary report/decisional support to cue referral (delivered to PCPs and care managers) and Job aid on mental health management	Prompts/cues	Environmental restructuring, education (*continued learning, adaptation, improvement*)	X	X	X	X	X
-In EPIC -ESDM Tool/brochure delivered by text/email/in-person by implementation staff/navigator Available every encounter Fidelity, feasibility, sustainability	-EPIC Smart phrase for treatment optimization -eSDM tool with prompt to PCP/care managers	Adding objects to environment	Environmental restructuring, enablement (*continued adaptation, improvement)*	X	X	X	X	
Zoom ID team meetings/emailed newsletter delivered by clinic mental health staff/administrators/implementation team At least quarterly Acceptability, feasibility, fidelity, sustainability	Feedback on screening, appropriate referral, attendance rates frequency, intensity, duration, and patient outcomes (clinic/care managers/providers)	Feedback on behavior and outcome(s) of the behavior	Education, persuasion, training (*continued organizational learning, evaluation, improvement)*	X		X	X	X
Clinic mental health staff/administrators/navigators At least quarterly Fidelity, feasibility, acceptability, sustainability	Triage patients based on eSDM tool input Screening support and education Quality improvement initiatives	Restructuring physical environment	Environmental restructuring, Enablement (*continued adaptation, improvement*)	X	X	X		X
Zoom ID team meetings delivered by clinic mental health staff/administrators/implementation team At least quarterly Acceptability, fidelity, sustainability	Review CC and DepCare implementation, brainstorm how to improve every 2–3 months, review key determinants of successful CC sustainability (e.g., how to identify and engage PCP champion, billing support, quality improvement), engages clinic directors/opinion leaders to provide feedback	Problem-solving, credible source	Enablement, Persuasion *(continued stakeholder engagement, problem-solving, learning, evaluation adaptation, improvement)*	X	X	X	X	X

Abbreviations: *CC* collaborative care; *DSF* Dynamic Sustainability Framework; *EPIC* Epic Systems Corporation electronic health record; *eSDM* electronic shared decision making; *PCP* primary care providers; *ID* intervention development

Key intervention functions for promoting ongoing behavior change that met all APEASE criteria and would support continuous learning/improvement (DSF) included environmental restructuring, education, training, modeling, persuasion, education, and enablement. *W*e determined that the most feasible multi-level strategy (with corresponding *behavior change techniques* and *ERIC strategies)* for promoting sustainable behavior change would center around a patient-level video-assisted electronic shared decision making (eSDM) web application to provide culturally targeted psychoeducation, motivational messaging from care managers and patients with lived experiences, and treatment preference matching/automated shared decision making preliminarily delivered in the waiting room to patients with elevated depressive symptoms prior to primary care visits to ensure equitable access to technology (*prompts/cues, information about consequences, credible source, verbal persuasion, and restructuring the environment; develop/distribute educational materials, model/simulate change, change physical structure and equipment*) (Table 2).

At the provider/system level, a preliminary strategy would involve yearly mental health/CC general medicine grand rounds as well as in-person delivery of DepCare tool summary reports to PCPs/care managers on treatment preferences/barriers in the waiting room at the time of a visit to support patient-provider communication, triage, and referrals (*prompts/cues and information about consequences; ongoing training, educational meetings, remind clinicians, audit and provide feedback*). Initially, no system-level strategy was planned but based on care manager meetings it became clear we would need local (as opposed to external NYS delivered) problem- solving meetings with researchers, care managers and PCPs to discuss contextual factors key to CC sustainability (e.g., billing education/support, cultural tailoring) (*problem-solving; provide local technical assistance, implementation meetings, learning collaborative*).

### Step 3 Results. Rapid Cycle user design process to operationalize and refine strategy components

Stakeholder characteristics are described in Additional File 4. Table 4 describes adaptations to the strategy components by the creative team (*n* = 4), intervention development team (*n* = 7), and advisory board (*n* = 11) categorized by usability/workflow themes and DSF/COM-B constructs targeted. We further describe ongoing CC adaptations and contextual factors.

**Table 4:** Rapid cycle, expert stakeholder adaptations to the DepCare multi-level implementation strategy to promote collaborative care sustainability

Theme	System/provider-level strategy adaptation (COM-B construct and DSF/contextual factor targeted)	Patient-level strategy adaptation (COM-B construct and DSF/contextual factor targeted)	Contextual factors affecting healthcare system depression screening/treatment/collaborative care program (2018–2021)
**Functionality (*usability, understandability)*** (perceived effectiveness, efficiency, and “ease- or lack-of-ease of use; information is comprehendible)	**1. Lessen cognitive load of Preference report** (e.g., use icons, pictures, pictograms, and colors give clear treatment support) (*capability*) – ID Team **2. Create onboarding video** (more accessible than Grand Rounds meeting) (*opportunity, capability, motivation*) – ID Team	**1. Effective patient activation language** "*It's up to you" is too difficult for a depressed patient..[change to] ‘your doctor is ready to hear from you, all you need to do is click here’ " (Motivation) –* Advisory Board **2. Flexible operability/delivery** (e.g., for those poor access to internet/devices provide devices in clinic; phone formatting, paper copy/brochure to write appointment reminders/goals) (*opportunity/practice setting, capability, motivation)* Advisory Board, Core Team **3. Use low literacy screeners, use voiceover** (e.g., PHQ 9) (*capability)* – ID team	**Practice setting** • Social determinants of health screening mandates (e.g., with iPad and navigators in the waiting room) • Electronic health record transitions to EPIC with need to align registries • Mental health provider/care managers turnover, 1 care manager covering multiple clinics • Inappropriate referrals to CC worsen “*Providers using CC as walk-in mental health clinic…Not their purpose & not everyone is guaranteed treatment…Providers too comfortable sending all patients to CC; not asking about depression, treatment in assessments resulting in inappropriate referrals ….CC still getting referrals when patients just need to have their medication titrated, should by* *handled by PCP”* • Depression screening done with e-check in process **Ecological system** • Office of Mental Health includes collaborative care reimbursement codes for anxiety (not just depressed patients) • Depression Screening becomes accountable care organization metric of interest in health care system (need to provide clinics with depression screening support, staff education) **Practice setting and ecological system** • Suicide screening mandates • Due to COVID-19, remotely delivered collaborative care leads to improvement in show rates but inappropriate referrals (need to focus on referral and depression treatment optimization not just initiation)
**Content and usefulness** (extent the tool and content/information provided by it) is perceived as helpful during clinical decision-making and care delivery)	**3. Add depression/suicide screening video for staff** (improve quality of screening in telemedicine era) (*capability*) – ID Team **4. Address post-COVID-19 barriers in problem solving meetings** (transition from billing/start up barriers with CC to telepsychiatry, leveraging EPIC, remote delivery, identifying champions) (*opportunity/Practice setting and Ecological System, capability*) – ID Team, Advisory Board **5. Adapt provider education to include motivational messaging, onboarding** (not just CC initiation/referral but triage, optimization, appropriate referrals, how to use DepCare tool) (*capability, motivation, opportunity*) – ID team **6. Summary report to focus on treatment optimization/medication support** (*opportunity/Practice setting, capability*) –Advisory Board **7. Add Job Aid and EPIC Smart phrase:** to support appropriate referral, decisional support (*capability*) – ID Team	**4. Patient activation/Education/Goal Setting** (less so on shared decision making, selecting options) “*Messaging should include language about – things can be different. You are taking a step to do something today. Even though this is scary, this could be the start of getting your life back. We're happy to be partners in your care”,* “*overall goal to get you to a care manager”* (*motivation, capability*) – ID team, Advisory Board, Creative Team **5. Triage/personalize experience** (based on history/treatment/severity, include atypical symptoms link anger/pain, options to defer treatment) *“messaging* *should emphasize that there’s no one correct model of treatment, that each person’s treatment is tailored to fit their needs.”* (*motivation, opportunity/Practice setting, capability*) – Creative Team, Advisory Board, Intervention Team **6. Add content hub** (links to suicide, wellness, and depression treatment resources and hotlines) (*opportunity/ecological system/practice setting, capability*)** –** Advisory Board **7. Add patient story** (psychoeducation, activation, cultural tailoring) (*motivation, capability*) **8. Include anxiety/suicide screening** (*Opportunity/practice setting and ecological system*) Advisory Board, ID team	
**Visibility, workflow, and navigation** (extent to which noticed or attended; general order/sequence of tasks and activities in patient encounter; ability to move through the system)	**8. Screening support/align with clinic initiatives** (e.g., include suicide screening, provide depression screening support if setting not screening, leverage screening results if clinic screening; provide quality improvement depression screening education video) (*Opportunity/Practice Setting, motivation, capability*) – Core Team, Advisory Board **9. Electronic preference report (**paper copy in person EPIC message to both PCP and care manager, email) (*Opportunity*) – ID Team **10. Option to bypass provider Ensure care managers receive tool results** *“infeasible to have a self-referral option because of the influx of patients” (opportunity/Practice setting, capability, motivation)* – ID Team	**9. Flexible delivery**. Medical assistants deliver to all PHQ2 positive patients options for waiting room admin with staff vs. text/email at home, bypass PHQ if already done by clinic (*opportunity/practice setting*) – Advisory Board, ID Team	

*CC* Collaborative care, *ID* Intervention development, *OMH* Office of Mental Health, *PHQ* Patient Health Questionnaire, *PCP* Primary care provider

#### CC program adaptations and key contextual factors

Strategy delivery was affected by competing initiatives (e.g., iPad delivered social determinants of health screening in waiting rooms), transition to *EPIC Systems Corporation* electronic health record (which disrupted referral and registry processes), care manager turnover, expanded CC reimbursement for anxiety not just depression, and transition to remote/hybrid CC delivery during COVID-19, which improved show rates but increased inappropriate referrals of severe cases. Few factors were addressed by external technical assistance provided by state officials.

#### Patient-level strategy adaptation

The initial (version 1) DepCare prototype consisted of depression screening (with the validated Patient Health Questionnaire (PHQ)-9), a depression “score report” (describing the meaning of their score), a treatment preference and barriers checklists, care manager motivational video, and assessment of patient’s interest in seeing a care manager and starting a medication. The tool was available in English and Spanish and took 10 to 15 min to complete based on digital literacy. Details of the iterative user-centered design process of the DepCare prototype with patients and providers as well as version-by-version adaptations will be described separately. Key adaptations included (1) personalizing based on patients’ depression severity/treatment history; (2) incorporation of suicide/anxiety screening; (3) strengthening triage functionality and connection to content hub/treatment resources to address care manager capacity concerns; (4) transition from treatment decisional support to education/patient activation to reduce no-show rates (*motivation, capability)* to triage/personalization/treatment optimization to address inappropriate referrals (*opportunity*); (5) reduced waiting room delivery, portal/multi-modal delivery [including paper versions to address literacy] (*opportunity/practice setting*, *capacity, motivation*); and (6) addition of patient video/story to facilitate cultural tailoring and activation (*capability, motivation*).

#### Provider-level strategy adaptation

The preliminary strategy included an in-person PCP summary report of patient preferences and yearly in-person general CC education. The PCP educational strategy transitioned to email-delivered short video/newsletters on CC indications and feedback on provider treatment optimization rates*.* Summary reports were delivered via EPIC/email and expanded to include care managers to better prepare for visits, triage, and avoid inappropriate referrals. We created an EPIC Systems Corporation smart phrase i.e., “dot phrases,” that allow commonly used chunks of text to easily be inserted into patient notes”).

#### System level strategy adaptation

Finally, we expanded the ongoing problem-solving meetings (focused on billing, cultural tailoring with local care managers) to include behavioral health providers across the institution to address local barriers and quality improvement (e.g., creating and disseminating quality improvement videos for medical assistant administered depression screening).

### Step 4 results: confirm behavior change techniques and finalize strategy

#### Confirm behavior change techniques in final materials

An external BCW expert reviewed all final adapted materials. The combination of the brochure and DepCare tool appropriately represented all the initially mapped behavior change techniques, except for restructuring of the physical environment, which was an inherent component of how the tool would be delivered. The external BCW expert mapped all behavior change techniques to the provider marketing video, except for *demonstration of the behavior* and *add* *objects to the environment*. We refined the provider video to better demonstrate how a provider would refer to CC and strengthened descriptions of how the summary report and smart phrase as additions to the environment. The expert appropriately identified most behavior change techniques related to the quarterly implementation team meetings, except for *feedback on the behavior, action planning,* and *credible source*. We removed action planning given lack of feasibility and opted to better differentiate behaviors (e.g., screening/referrals) from outcomes of behaviors (e.g., clinic-level depression symptom burden) during meetings/newsletters. We further engaged care managers/clinic administrators to lead meetings and send newsletters as credible sources. If proven effective in our ongoing trial, links to all materials will be made widely available.

#### Final implementation strategy for CC sustainability

The final multi-level implementation strategy is presented in Table 5. The patient-level centers around implementation team/staff-delivered (email/text/in-person based on patient preference) DepCare tool (IR CU19184), which includes enhanced depression and anxiety screening, diagnosis recognition support, patient activation, personalized psychoeducation, patient/care manager videos promoting patient treatment engagement, personalized medication selection support and link to external treatment. The *provider-level* strategy includes administrator-delivered email of educational/motivational video on CC and optimal management of depression and comorbid anxiety, invitations to problem-solving/technical assistance meetings, and automatically generated DepCare tool decisional support on individual patient treatment preferences delivered to both the provider and care managers. The *clinic-level* strategy includes quality improvement support and education around valid depression screening as well as local technical support/problem solving for mental health staff/providers co-lead with implementation team members. In the last advisory board meeting (*n* = 4) prior to launch, the mean appropriateness of the intervention based on the validated scale was 4.56 and the mean feasibility was 4.36.

**Table 5:** Final multi-level multi-component DepCare implementation strategy for sustaining CC

	**Experimental arm**	**Enhanced usual care arm**
Clinic	(1)Support and education around valid depression and anxiety screening (2) Local technical assistance for collaborative care program	(1) Support and education around valid depression and anxiety screening (2) Local technical assistance for collaborative care program
Provider	(3) One-time presentation or video with education and motivational messaging around collaborative care, functionality of the DepCare patient tool, and optimal management of depression and comorbid anxiety (4) 2–4 quality improvement/implementation team meeting per year on optimizing mental health treatment in primary care and DepCare strategy (i.e., multi-level, multi-component intervention) implementation (5) Automatically generated decisional support on individual patient treatment preferences (i.e., for every patient who receives the DepCare patient tool)	(3) Usual care (social workers are notified of suicidal patients)
Patient	(6) Patient tool comprised of enhanced depression and anxiety screening (includes option for voice-over questions, point-and-click responses), and for those who screen positive for depressive symptoms (with or without comorbid anxiety), diagnosis recognition support, psychoeducation, videos promoting patient engagement in treatment, and personalized medication selection support	(4) Usual care (patients are intermittently screened for depression/anxiety based on clinic resources or provider indications)

## Discussion

Using CC sustainability as a use case, we apply the DSF, BCW, and user-centered design principles to provide a multi-step roadmap for designing implementation strategies that promote sustainability, which involves identifying key “sustainability behaviors” and their barriers, selecting behavioral theory-informed strategies for sustainability, and finally operationalizing and adapting these strategies using rapid cycle, multi-stakeholder processes (Fig. 1). We provide a framework for incorporating more behavioral science into sustainability efforts. We go on to demonstrate how user-centered design principles can be used to operationalize and adapt implementation strategies to address fluctuations in contextual factors. For this use case, we found that a multi-level, multi-component strategy centered around an electronic automated shared decision-making, triage and referral tool (DepCare), provider education/activation/decision support and ongoing problem-solving meetings would be the most acceptable and equitable strategy for supporting multi-level behaviors and facilitating continued improvement/training processes.

Our study adds to the sustainability literature in several ways. First, experts have conceptualized sustainability as a dynamic construct that allows adaptation in response to contextual influences and organizational/implementer/intervention characteristics, calling for more research to identify and evaluate planned strategies to support sustainability in real world settings [18]. A recent systematic review of strategies that promote sustainability found few studies that employed a conceptual framework to guide in strategy development and highlighted key sustainability barriers (i.e., limited funding/resources) and facilitators (i.e., need for adaptation/alignment and funding) [38]. Our study, informed by DSF, answers calls for theory-informed, real world sustainability evaluation studies and proposes methods for operationalizing and tracking continued adaptations/improvements/evaluations not just of EBIs but strategies themselves via user-centered design principles. Experts have increasingly remarked on the need to consider user-centered design for improving the fit between evidence-based practices and implementation context and that the two fields can be complementary [22]. However, there are few use cases for integrating the two disciplines, particularly as it relates to sustainability. We posit that user-centered design may also be one method for facilitating ongoing adaptations, refinements, and improvements of implementation strategies integral to sustainability [19]. Integral to the process of sustainability is stakeholder engagement [18], and we describe ways in which stakeholders should be involved not only in understanding successes and failures of early implementation strategies but in developing, refining, and delivering strategies for sustainability.

Second, we propose a multi-step process for operationalizing the DSF to incorporate behavior change theory more explicitly to ongoing quality improvement/adaptation processes. In illustrative applications of the DSF, Chambers et al. note that care management is “influenced by drivers at patient, provider, organization and system levels”, requiring coordination among multiple stakeholders and continued assessment of fit over time, [19] all of which we posit necessitates sustained multi-level behavior change. Implementation experts recommend choosing frameworks that fit sustainability needs [39] and not every sustainability effort will warrant a behavioral lens. We identified ongoing behavior change as a key component of CC sustainability, which will require strategies that address both local contextual factors and behavioral constructs (i.e., capability, opportunity, motivation) of several CC end users. The BCW has been extensively used to design behavioral interventions and develop implementation strategies [26, 33, 40]. To our knowledge, we are among the first to apply a sustainability lens to the BCW by prioritizing behavior change techniques and implementation strategies that are not just acceptable/equitable/feasible but adhere to tenets of dynamic sustainability (i.e., continued education/improvement). Systematically incorporating behavior change techniques like feedback of behavior, prompts/cues, and problem solving may improve the efficiency and effectiveness of dynamic sustainability processes. We also demonstrate the ways in which usability and behavioral factors (in addition to ecological and local contextual factors) may drive adaptations to both interventions and strategies.

Finally, our proposed roadmap is adaptable. A recent mixed methods study also found that several elements of the Consolidated Framework for Implementation Research (CFIR) (e.g., ongoing coalitions, networks and partnerships, infrastructure and capacity to support sustainability, community need for programs, ongoing evaluation of performance and outcomes) are integral to the sustainability of evidence-based mental health and behavioral interventions [41]. Other use cases may find that incorporating CFIR constructs is warranted during Phase 1 of our roadmap. We posit that attention is also needed to maximally support optimal behaviors and thus long-term fidelity. Nonetheless, further work is needed to rigorously validate conceptual and methodological aspects of sustainability [18], including our behavioral approach. Future work will establish whether our strategy resulted in improved sustainability indices, including treatment optimization rates in settings seeking to sustain CC (both those with and without external implementation strategy support). Our ongoing work will also elucidate the utility of adapting our DepCare strategy to other implementation use cases (i.e., depression screening and treatment in heart disease patients) [42].

Overall, extensive research on CC implementation concludes that external support is essential for real-world effectiveness [14]. Centralized (policy-level) strategies for CC implementation (i.e., external technical assistance, reimbursement, quality monitoring) often focus on care manager behavior and CC quality/fidelity/implementation processes [16]. While these strategies inherently improve some sustainability indices, the premise of our study is that sustainability will also require ongoing behavior change, including continued care manager delivery of CC but also provider referral/optimization and patient treatment initiation/persistence. For example, late adopting CC patients may have more treatment-resistant, complex, comorbid mental health conditions. Researchers have also long demonstrated suboptimal mental health engagement/competency/optimization by providers [43, 44], further impacting implementation outcomes [45–48]. Strategies not attuned to local contextual factors and related multi-level behavior change may lose ground in the sustainability phase. Our theory-informed, multi-level approach to targeting behaviors key to sustainability suggests the need for ongoing problem-solving meetings, patient activation/educational tools (that also target contextual factors by triaging patients, saving providers time, and connecting to treatment) and ongoing provider-level decisional support/education to support multi-level engagement.

There were several limitations to our study. Despite our incorporation of DSF tenets, our use of the multi-step BCW (which is often more static than dynamic) may have failed to identify key contextual determinants of and adaptative strategies for sustainability [38, 41]. We also focused on adapting the strategy itself while tracking adaptations to CC in response to contextual factors. Sustainability may require more intensive adaptations to EBIs themselves. Relatedly, ERIC strategies and the BCW have yet to be tested or validated for sustainability. Instead, our roadmap should be interpreted as an adjunctive process for incorporating more behavioral science into sustainability efforts. Since study completion, newer frameworks for tracking adaptations of interventions and strategies have also emerged, which may have allowed us to better operationalize our process [49]. While our adaptation process innovatively utilized user-centered design procedures and multiple stakeholders to adapt (not just track adaptations), this was an intensive process and may not be replicable. In all, it took 30 months to complete due to the COVID-19 pandemic. In addition, the adaptation of the implementation strategy occurred in experimental (vs. real time) settings within a subset of individuals, which did not allow us to evaluate the effects of each strategy adaptation on validated sustainability measures (other than usability and perceived feasibility). Finally, we did not consider development costs when deciding on strategy components, though concluded that a freely available web application had the most potential to be sustainably delivered (e.g., sent to all patients on waiting/screen positive lists by care managers/care coordinators while other strategy components were already delivered by the healthcare system (e.g., problem-solving meetings, provider education). Nonetheless, an inherent consideration within implementation science is that strategies (including those that promote sustainability) need to themselves be sustained.

## Conclusion

Our study provides a rigorous, multi-step process for applying behavioral science, implementation science, and user center design to select and adapt implementation strategies to fit dynamic local contexts and sustain interventions. Our strategy is currently being tested in settings with collaborative/integrated care, during which we will track further adaptations to both the strategy and evidence-based intervention and assess the effects of our multi-level strategy on treatment optimization in clinics seeking to sustain CC. This study has marked implications to developing, adapting, and testing strategies that specifically promote sustainability in a broad range of populations and settings.

## Supplementary Information

**Additional file 1.** The behavior Change Wheel.

**Additional file 2.** Definitions of Intervention Functions and Policy Categories: The Behavioral Chance Wheel.

**Additional file 3.** Using the Behaviour Change Wheel to operationalizing multi-level behaviors around sustaining collaborative care.

**Additional file 4.** Expert Stakeholder Characteristics.

## Data Availability

The datasets used and/or analyzed during the current study are available from the corresponding author on reasonable request.

## Abbreviations

ACN: Ambulatory Care Network
BCW: Behaviour Change Wheel
CC: Collaborative Care
CFIR: Consolidated Framework for Implementation Research
COM-B: Capability, Opportunity, Motivation – Behavior Model
COVID-19: Coronavirus disease 2019
DSF: Dynamic Sustainability Framework
ERIC: Expert Recommendations for Implementation Change
NYS DOH: New York State Department of Health
NYS OMH: New York State Office of Mental Health
PHQ: Patient Health Questionnaire
