# Supplementary material for: A theory-informed, rapid cycle approach to identifying and adapting strategies to promote sustainability: optimizing depression treatment in primary care clinics seeking to sustain collaborative care (The Transform DepCare Study)
Source: Implement Sci Commun. 2023 Jan 25;4:10. doi: 10.1186/s43058-022-00383-2 (PMC9875183; doi:10.1186/s43058-022-00383-2)
Supplement: Supplementary file 1 — Additional file 1. The behavior Change Wheel. [file 43058_2022_383_MOESM1_ESM.docx]

SUPPLEMENTARY MATERIAL

Additional File 1. The behavior Change Wheel


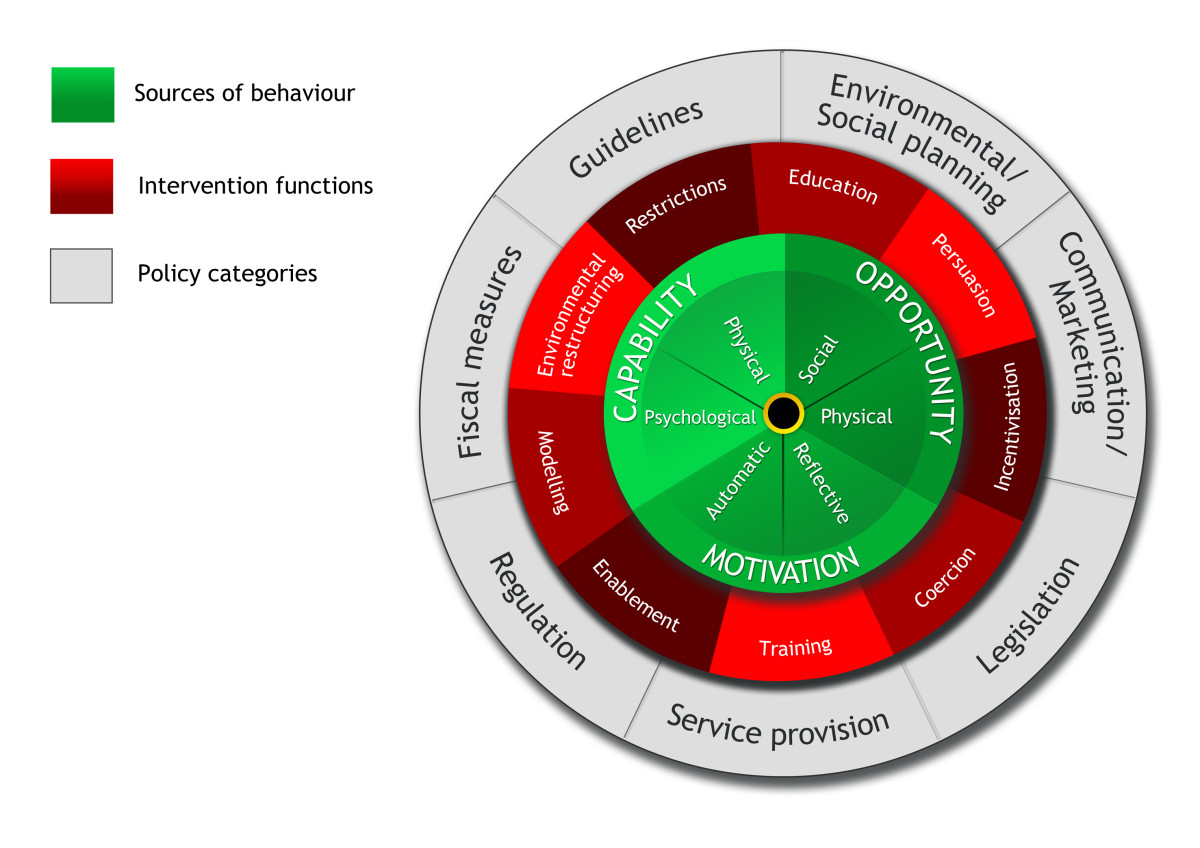


**A**ffordability

**P**racticability

**E**ffectiveness

**A**cceptability

**S**afety

**E**quity
