# Supplementary material for: A theory-informed, rapid cycle approach to identifying and adapting strategies to promote sustainability: optimizing depression treatment in primary care clinics seeking to sustain collaborative care (The Transform DepCare Study)
Source: Implement Sci Commun. 2023 Jan 25;4:10. doi: 10.1186/s43058-022-00383-2 (PMC9875183; doi:10.1186/s43058-022-00383-2)
Supplement: Supplementary file 2 — Additional file 2. Definitions of Intervention Functions and Policy Categories: The Behavioral Chance Wheel. [file 43058_2022_383_MOESM2_ESM.docx]

**Additional File 2. Definitions of Intervention Functions and Policy Categories: The Behavioral Chance Wheel.** Checkmarks signify policy categories that correspond to each intervention function in the BCW. Those intervention functions and policy categories shaded in dark gray signify that all APEASE (affordability, practicality, efficacy, acceptability, safety, equitable) criteria were met at both the patient and provider/system levels. Light gray is provider/system-level only.

|  | | **INTERVENTION FUNCTIONS** | | | | | | | | |
| --- | --- | --- | --- | --- | --- | --- | --- | --- | --- | --- |
|  |  | **Education**  Increasing knowledge/ understanding | **Persuasion**  Using communication to induce pos/neg feelings or stimulate action | **Incentivi-sation** Creating an expectation of reward | **Coercion** Creating an expectation of punishment or cost | **Training**  Imparting Skills | **Restriction**  Using rules to reduce opportunity to engage in target behavior (reducing the opportunity to engage in competing behaviors) | **Environmental restructuring**  changing the physical or social context | **Modeling**  Providing an example for people who aspire to or imitate | **Enablement**  Increasing means/reducing barriers to increase capability (beyond education) or opportunity (beyond environmental restructuring) |
| **POLICY CATEGORY** | **Communication/**  **Marketing:** Using print, electronic telephonic or broadcast media | √ | √ | √ | √ |  |  |  | √ |  |
|  | **Guidelines:** Creating documents that recommend or mandate | √ | √ | √ | √ | √ | √ | √ |  | √ |
|  | **Fiscal:** Using the tax system to reduce or increase the financial cost |  |  | √ | √ | √ |  | √ |  | √ |
|  | **Regulation:** Establishing rules or principles of behavior or practice | √ | √ | √ | √ | √ | √ | √ |  | √ |
|  | **Legislation:** Making/changing laws | √ | √ | √ | √ | √ | √ | √ |  | √ |
|  | **Environmental/ social planning:** Designing and/or controlling physical /social environment |  |  |  |  |  |  | √ |  | √ |
|  | **Service Provision:** Delivering a service | √ | √ | √ | √ | √ |  |  | √ |  |
