# Supplementary material for: A theory-informed, rapid cycle approach to identifying and adapting strategies to promote sustainability: optimizing depression treatment in primary care clinics seeking to sustain collaborative care (The Transform DepCare Study)
Source: Implement Sci Commun. 2023 Jan 25;4:10. doi: 10.1186/s43058-022-00383-2 (PMC9875183; doi:10.1186/s43058-022-00383-2)
Supplement: Supplementary file 3 — Additional file 3. Using the Behaviour Change Wheel to operationalizing multi-level behaviors around sustaining collaborative care. [file 43058_2022_383_MOESM3_ESM.docx]

**Additional File 3. Using the Behavioral Change Wheel to operationalizing multi-level behaviors around sustaining collaborative care**

|  | **System** | **Provider** | **Patient** |
| --- | --- | --- | --- |
| **Target Behavior** | Sustain CC | Optimize Depression treatment | Engage in depression treatment |
| **Who Needs to perform the behavior** | Staff  Care managers/psychiatrists | Provider | Patients with elevated depressive symptoms |
| **What do they need to do differently to achieve desired change?** | Screen for depression; Deliver CC (treat to target, registry-based treatment with psychiatry consultation) | Titrate/Initiate/Add antidepressant, refer for initial/additional collaborative care/psychotherapy and/or counsel on adherence | Pick up an antidepressant and/or attend visit with care manager/psychiatrists/psychologists |
| **When do they need to do it?** | At each visit | At primary care visit | Following their primary care appointment |
| **Where do they need to do it?** | In clinic | In clinic | In clinic or via Telemedicine/home |
| **How often do they need to do it?** | Daily | At each visit | Every time they have elevated depressive symptoms |
| **With whom do they need to do it?** | Providers and patients | Patients with elevated depressive symptoms | Primary care and/or behavioral providers |
