# Supplementary material for: A theory-informed, rapid cycle approach to identifying and adapting strategies to promote sustainability: optimizing depression treatment in primary care clinics seeking to sustain collaborative care (The Transform DepCare Study)
Source: Implement Sci Commun. 2023 Jan 25;4:10. doi: 10.1186/s43058-022-00383-2 (PMC9875183; doi:10.1186/s43058-022-00383-2)
Supplement: Supplementary file 4 — Additional file 4. Expert Stakeholder Characteristics. [file 43058_2022_383_MOESM4_ESM.docx]

**Additional File 4. Expert Stakeholder Characteristics**

| Team | Gender | Specialty |
| --- | --- | --- |
| Advisory Board | Female | Biomedical Informatics |
| Advisory Board | Female | Psychiatry |
| Advisory Board | Female | Internal Medicine/Clinic Director |
| Advisory Board | Female | Cardiology/Clinic Director |
| Advisory Board | Male | Psychiatry |
| Advisory Board | Female | Psychology/Patient Experience |
| Advisory Board | Male | Internal Medicine/Shared Decision-Making Researcher |
| Advisory Board | Female | Psychiatry/Collaborative Care Researcher |
| Advisory Board | Male | Internal Medicine/Digital Mental Health Researcher |
| Advisory Board | Male | Behavioral Medicine/Statistics |
| Advisory Board | Male | Biomedical Informatics |
| Intervention Development | Female | Social Work/Behavioral Collaborative Care Leadership |
| Intervention Development | Male | Social Work/Depression Care Manager |
| Intervention Development | Female | Internal Medicine/Behavioral Trial Researcher |
| Intervention Development | Male | Psychiatry/Behavioral Activation |
| Intervention Development | Female | Internal Medicine/Clinic Director |
| Intervention Development | Female | Social Work/Depression Care Manager |
| Intervention Development | Female | Social Work/Depression Care Manager |
| Intervention Development | Female | Patient Stakeholder |
| Creative Team | Male | Creative Director |
| Creative Team | Female | Production/Content |
| Creative Team | Female | Design Expert |
| Creative Team | Male | Developer |
